# Identifying the Anti-MERS-CoV and Anti-HcoV-229E Potential Drugs from the *Ginkgo biloba* Leaves Extract and Its Eco-Friendly Synthesis of Silver Nanoparticles

**DOI:** 10.3390/molecules28031375

**Published:** 2023-02-01

**Authors:** Ezzat H. Elshazly, Alyaa Nasr, Mohamed E. Elnosary, Gamal A. Gouda, Hassan Mohamed, Yuanda Song

**Affiliations:** 1 Department of Botany and Microbiology, Faculty of Science, Al-Azhar University, Assiut 71524, Egypt; 2 Department of Botany and Microbiology, Faculty of Science, Menoufia University, Shebin El-Kom 32511, Egypt; 3 Department of Botany and Microbiology, Faculty of Science, Al-Azhar University, Nasr City 11884, Egypt; 4 Department of Chemistry, Faculty of Science, Al-Azhar University, Assiut 71524, Egypt; 5 Colin Ratledge Center for Microbial Lipids, School of Agricultural Engineering and Food Science, Shandong University of Technology, Zibo 255000, China

**Keywords:** anti-coronavirus, silver nanoparticles, *Ginkgo biloba*, natural products

## Abstract

The present study aimed to estimate the antiviral activities of *Ginkgo biloba* (GB) leaves extract and eco-friendly free silver nanoparticles (Ag NPs) against the MERS-CoV (Middle East respiratory syndrome-coronavirus) and HCoV-229E (human coronavirus 229E), as well as isolation and identification of phytochemicals from GB. Different solvents and high-performance liquid chromatography (HPLC) were used to extract and identify flavonoids and phenolic compounds from GB leaves. The green, silver nanoparticle synthesis was synthesized from GB leaves aqueous extract and investigated for their possible effects as anti-coronaviruses MERS-CoV and HCoV-229E using MTT assay protocol. To verify the synthesis of Ag NPs, several techniques were employed, including X-ray diffraction (XRD), scan, transmission electron microscopy, FT-IR, and UV–visible spectroscopy. The highest contents of flavonoids and phenolic compounds were recorded for acetone, methanol, and ethanol as mixtures with water, in addition to pure water. HPLC flavonoids were detected as apegenin, luteolin, myricetin, and catechin, while HPLC phenolic compounds were pyrogallol, caffeic acid, gallic acid, and ellagic acid. In addition, our results revealed that Ag NPs were produced through the shift from yellow to dark brown. TEM examination of Ag NPs revealed spherical nanoparticles with mean sizes ranging from 5.46 to 19.40 nm and an average particle diameter of 11.81 nm. A UV–visible spectrophotometric investigation revealed an absorption peak at λ max of 441.56 nm. MTT protocol signified the use of GB leaves extract as an anti-coronavirus to be best from Ag NPs because GB extract had moderate anti-MERS-CoV with SI = 8.94, while had promising anti-HCov-229E, with an SI of 21.71. On the other hand, Ag NPs had a mild anti-MERS-CoV with SI = 4.23, and a moderate anti-HCoV-229E, with an SI of 7.51.

## 1. Introduction

*Ginkgo biloba* leaf (GB) is a perennial dioecious tree; also known as the maidenhair tree, it is native to China and may be introduced to other world regions. The leaves of GB are green in the summer, yellow in the fall, and fall off during the winter. The green flowers appear in the spring with a pleasant fragrance, but the plant fruit usually has a bad smell. The *Ginkgo biloba* leaf is a conventional medicinal and food supply containing various chemical elements such as polyphenols, alkylphenols, terpenoids, flavonoids, organic acids, distinctive flavones, etc. [1,2,3]. These compounds have a variety of positive actions in the human body, including foraging free radicals, lowering oxidative stress, reducing platelet aggregation, and serving as antitumor and antiaging agents [4]. These components can also affect how metals are converted into metal ions, forming complexes and controlled dimensions and forms of the resultant metal NPs. Alternatively, extracts from *Ginkgo biloba* leaves have been employed to stabilize gold [4] in the biosynthetic process, copper [5], grapheme [6], and silver [7] nanoparticles.

Nowadays, metal and metal oxide nanoparticles show therapeutic potential for various ailments due to their physiochemical characteristics [8,9]. Among the transitional elements, silver nanoparticles (Ag NPs) are a novel nanometal particle form widely incorporated in biological, therapeutic, and engineering disciplines [10]. In particular, synthetic biologically active Ag NPs have demonstrated substantial therapeutic aptitudes, including broad-spectrum antimicrobial, anticancer, anti-inflammatory, antioxidative, and antidiabetic action [11,12]. A modern review of the synthesis of Ag NPs and their uses in antimicrobial textile fabrics, food packaging films, and wound dressings is provided by Chengzhu L. et al. The antibacterial activity and cytotoxic effect in mammalian cells are given special consideration [13]. Typically, Ag-NP-containing biomedical products are used to treat tumors by rapidly degrading infected cells [14] and to prevent bacterial infections by hastening wound healing [15,16,17,18]. Ag NPs work well as anticancer drug carriers for the HeLa cell line when combined with doxorubicin and alendronate [19]. For mice given oral administration of Ag NPs, the liver and kidney are the primary target organs in various applications [20,21]. These organs are essential for the elimination of foreign chemicals. For three days, Bergin et al. gave Black-6 mice oral gavage with dosages of 0.1, 1, and 10 mg/kg of CT- and PVP-Ag NPs with diameters of 20 and 110 nm [22]. Following oral delivery, over 70.5–98.6% of the supplied Ag NPs were eliminated in feces. Thus, treated mice showed no toxicity and considerable tissue Ag NPs buildup.

The submicroscopic viruses are infectious agents that can only multiply within an organism’s live cells. They can cause serious diseases, which may cause the death of humans and other creatures [23,24]. According to recent statistics, viruses are thought to be responsible for 2,000,000 human fatalities annually throughout the world [25]. SARS-CoV), MERS-CoV, and SARS-CoV-2 have all occurred in the world [26,27]. The highly pathogenic MERS-CoV was initially discovered in the KSA in 2012 [28]. There were 27 countries, 1905 confirmed MERS-CoV cases, and 677 deaths as of 10 February 2017. (http://www.who.int/, accessed on 3 May 2022). Trials for MERS-CoV vaccines and therapies are continuously being conducted [28,29]. The common cold, which is often characterized by nasal congestion, rhinorrhea, sore throat, cough, and sneezing that may be conveyed by fever, is frequently linked with the human COVID-229E virus (HCoV-229E). According to statistics for HCoV-229E, symptoms usually peak on days three or four of the disease and disappear on their own. These viruses are less associated with respiratory infections, including bronchiolitis and pneumonia [30]. The recent focus in this range is on improving vaccines and medicines. Therefore, there is a continuous need for detailed exploration to recognize the characteristic patterns of this class of contagious agents. The silver nanoparticles have activity against numerous viruses such as HIV-1, hepatitis, respiratory syncytial, herpes simplex, monkeypox, H1N1 influenza A, and coronavirus, and have been investigated [31,32]. Ag NPs antiviral performance is based on the physical inhibition of binding among the virus and the host cell [31,32,33,34]. 

The purpose of this study is the exploration of *Ginkgo biloba* in a trial to discover novel medicines from its leaf extracts, as well as the identification of related secondary metabolites, along with the study of the characteristics of its green synthesis silver nanoparticles, which could be used as anti-coronaviruses (MERS-CoV and HCoV-229E). 

## 2. Results and Discussion

### 2.1. HPLC Analysis of Ginkgo Leaves Extract

Plant extracts are complex assortments of which the therapeutic effects are often ascribed to collective or synergistic effects of many components. HPLC analysis can help to explore such plant products [35]. It has been reported that extracts of Ginkgo leaves contain main chemical groups such as phenolics, terpenoids, and flavonoid glycosides [36]. The plant leaves also contain unique Ginkgo biflavones, alkylphenols, and polyprenols [1,37]. In the current study, the total contents of both flavonoids and phenolics in GB extract were abundant (Table 1) and recorded higher values than in a previous study by Ražna et al. [38]. We suggested that this could be attributed to the two studies’ different geographical localities of collected GB samples. The acetone–water extract contained the highest amount of total flavonoids and phenolics; this was achieved by Kobus et al. [39], who proved that aqueous acetone extracts from GB leaf exhibited an intense antioxidant activity in various in vitro model systems. 

**Figure 1:**
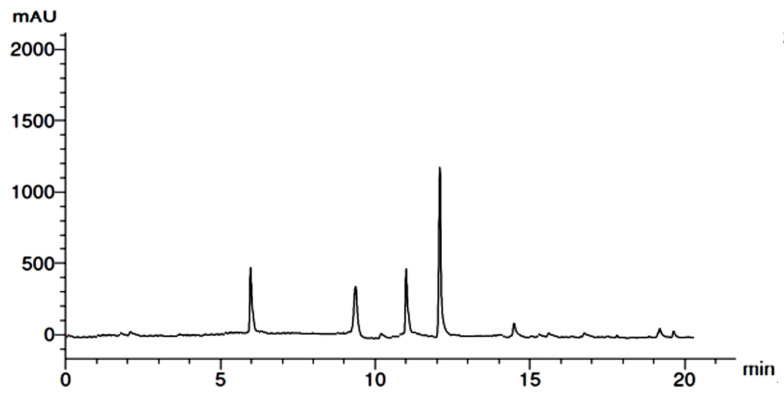
HPLC chromatogram of flavonoids from GB leaves.

**Figure 2:**
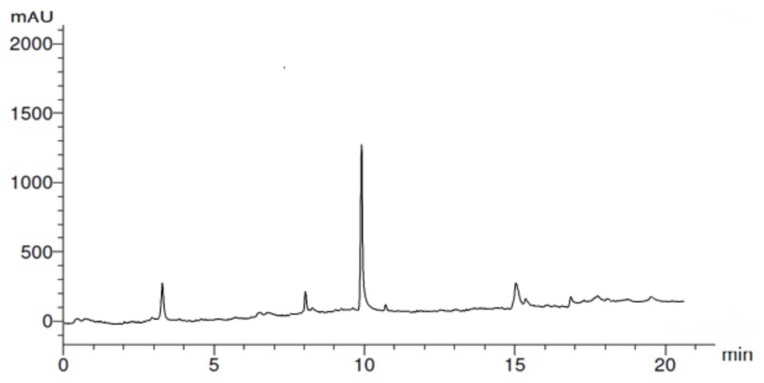
HPLC chromatogram of phenolics from GB leaves.

**Figure 3:**
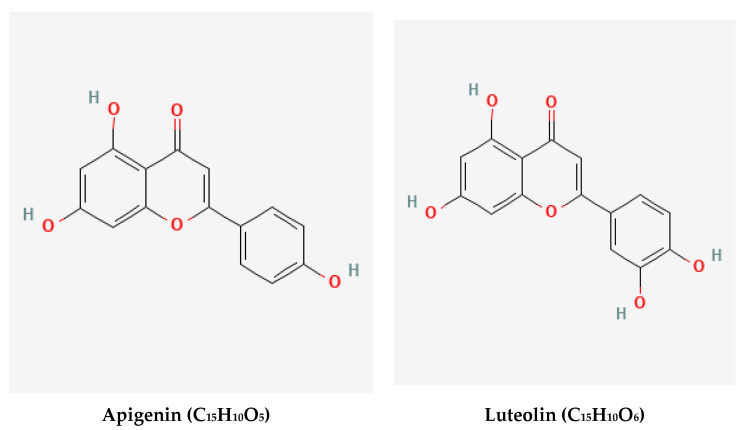

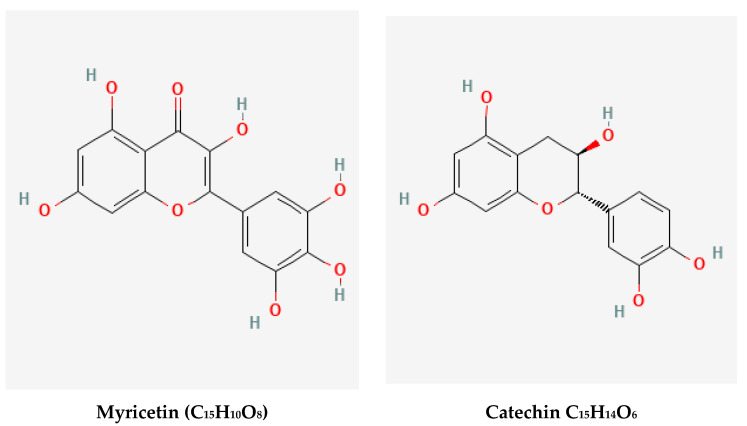
Chemical structures of the HPLC-detected flavonoids in GB leaves (https://pubchem.ncbi.nlm.nih.gov), accessed on 15 April 2022.

**Figure 4:**
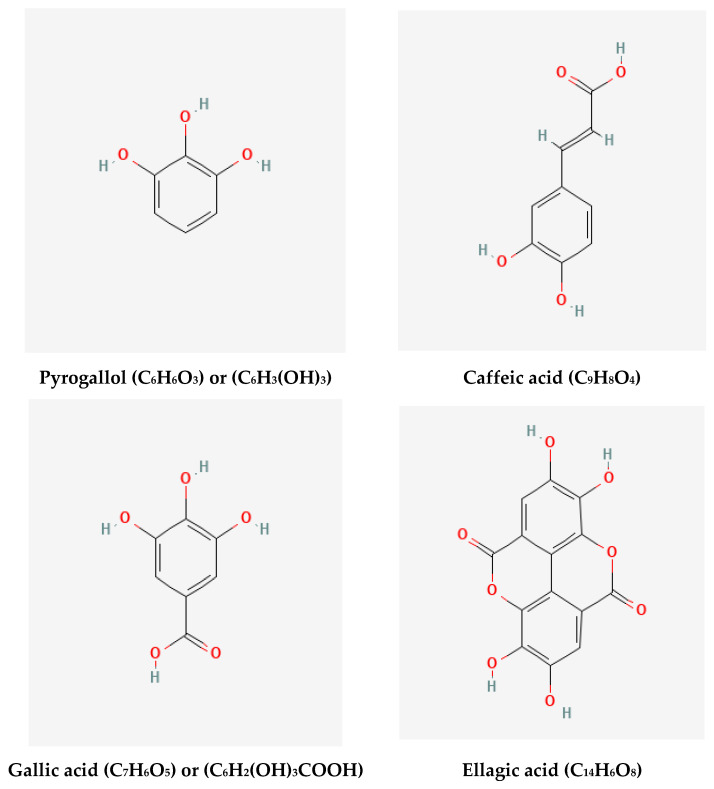
Chemical structures of the HPLC-detected phenolics in GB leaves (https://pubchem.ncbi.nlm.nih.gov), accessed on 15 April 2022.

In HPLC examination, flavonoid and phenolic components (Figure 1 and Figure 2) were detected in the extracts of GB leaves, such as apeginin, luteolin, and phenolic acids (Table 2 and Table 3). Their chemical structures are represented in Figure 3 and Figure 4. These outcomes are consistent with previous studies. The existence of more than 30 flavonoids has been described in Ginkgo extracts [40,41]. In addition, some flavonoid aglycones were considered in 13 GB extracts by LC-MS and HPLC-DAD [42].

Although kaempferol was reported before [43], it was not detected here. Formerly, Cheng et al. [44] found that many factors, such as plant age, climatic conditions, photosynthesis, type of fertilizers, and ecological dynamics, control flavonoid content in GB leaves. Commonly, light and temperature are the most critical ecological impacts; however, phenolic compounds were less investigated in GB leaves. Some phenolic acids, such as vanillic, para-coumaric, caffeic, isovanillic, sinapic, and ferulic, were identified in GB leaf extract [45]. 

Flavonoids are the primary bioactive ingredients of the GB leaves extracts, and they act as antioxidants and compounds with excellent stability and minimal toxicity that are antineuroexocytotic [46,47]. The beneficial effects of flavonoids in avoiding metabolic syndrome at various stages, including early-stage Alzheimer’s disease and cardiovascular disease, have been described [48]. Plants rely heavily on phenolic acids since they participate in many different processes, including allelopathy, enzymatic activity, photosynthesis, protein synthesis, and food intake, and due to their role as critical antioxidant molecules in all living cells [49]. 

### 2.2. UV–Vis Analysis 

**Figure 5:**
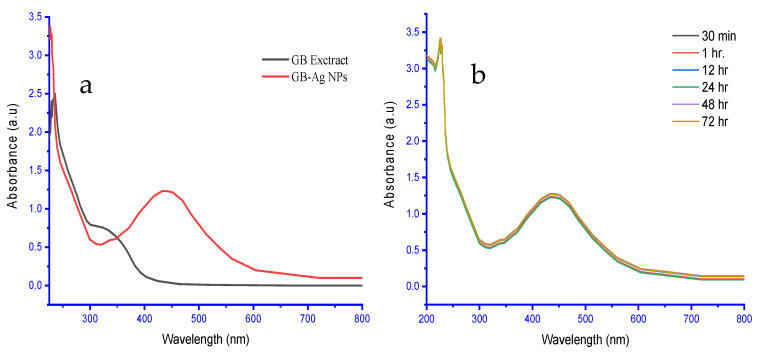
The UV–Vis spectroscopy of (**a**) the GB extract and as-biofabricated Ag NPs and (**b**) Ag NPs show good stability at regular intervals (for 3 days).

The spectrum analysis (UV–vis) of biosynthetic Ag NPs revealed a peak at 441.56 nm wavelength (Figure 5a) within the specified range of Ag NPs and indicated their presence in the reaction mixture [50,51]. The biosynthetic Ag NPs using GB leaf extracts were successfully carried out, as the change in the color of the reaction medium was light yellow to brown due to the reduction of silver nitrate aqueous solution. According to Figure 5b, it was confirmed that Ag NPs are stable over time in an aqueous reaction mixture. According to a spectral examination, the Ag NPs’ absorption maxima at 441.56 nm were constant for 30 min to 72 h. 

### 2.3. XRD Analysis

**Figure 6:**
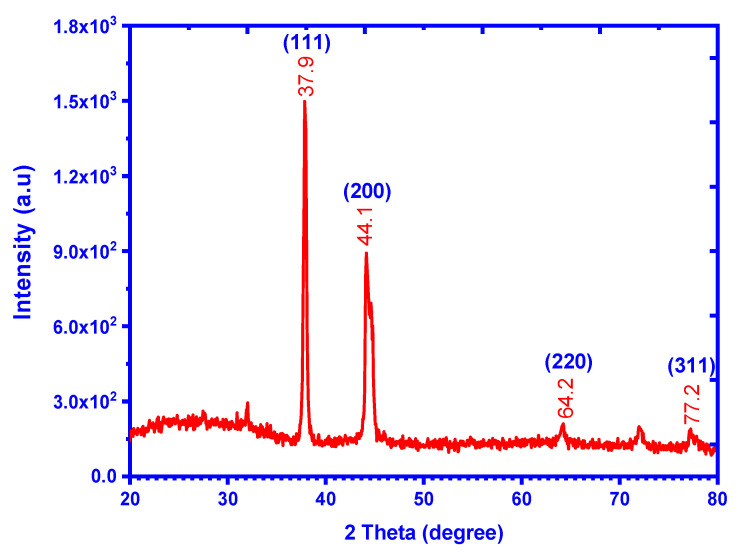
The as-biofabricated Ag NPs X-ray diffractogram from the GB leaf extracts.

The X-ray powder diffraction is a rapid and excellent technique to identify the material nature (whether it is crystalline or amorphous) at the nanoscale. In addition, XRD is beneficial for investigating the crystallographic system using comparison with the reference material cards. In Figure 6, the XRD pattern of the Ag NPs shows four principle peaks dedicated to the face-center spherical system of the Ag NPs material according to reference card JCPDS file No. 04-0783 [51,52]. Miller’s indices (h k l) were evaluated according to their two-theta degree values, and they were found to be (1 1 1), (2 0 0), (2 2 0), and (3 1 2), corresponding to two-theta of values 37.9, 44.1, 64.2, and 77.2°, respectively. Thus, the XRD confirms the crystalline phase of the Ag NPs, and from the angle value, it is clear that the compound is stable [53,54,55]. The calculated average size of Ag NPs by the XRD line broadening method [56] was ~11.9 nm.

### 2.4. FT-IR Analysis

**Figure 7:**
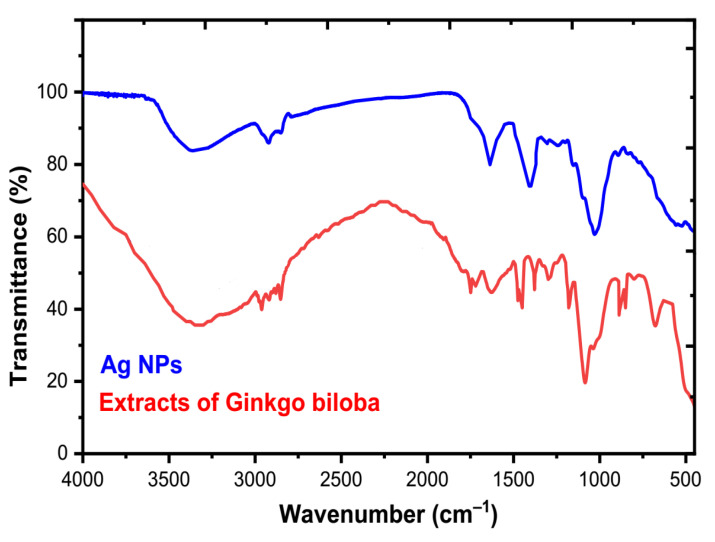
FT-IR spectra of the GB extract and as-biofabricated Ag NPs.

The significant role of FT-IR analysis was identified in confirming the participation of various functional groups in the capping stabilizing action of Ag NPs biosynthesis. In addition, other researchers reported that the content of the dried mass of most powder extracts is bioactive constituents such as flavonoids, terpenoids, phenolics, glycosides, and ginkgolides in their investigations [1,3]. Most bands in the GB extract were missing or deviated after the bioreduction procedure compared to before the reduction reaction [6,57]. This can be attributed to the bioactive molecules such as phenolics, flavonoids, terpenoids, and ginkgolides that are present in the GB extract that is involved in the bioreduction process. In Figure 7, FT-IR of GB leaf extract shows the presence of –CH stretching vibrations of –CH_3_ or –CH functional groups at 2958 cm^−1^ [58], O–H stretching at 3390 cm^−1^, C–O stretching and carboxyl C=O at 1378 cm^−1^ and 1720 cm^−1^, respectively, alkylphenols (1078 cm^−1^), and aromatic C=C (1632 cm^−1^) [5,59,60,61,62,63]. The peaks at 1632 and 1638 cm^−1^ were for O–H, C–O, and C=O as functional groups of GB extract. After the reduction process, the peaks shifted to a lower wave number side, such as 1619, 1378, 1045, 3158, and 2922 cm^−1^. The reduction process and capping of Ag^+^ into Ag^0^ NPs in the analysis may be due to organic acids, favonolds, and alkylphenols [64,65]. The O–H (stretching) in –COOH (vibration) in Ag NPs shifted from 3881 to 3390 cm^−1^. Plant extracts were used to demonstrate the water-soluble role of flavonoids in the reduction process of metal ions [66,67]. 

### 2.5. SEM Analysis

**Figure 8:**
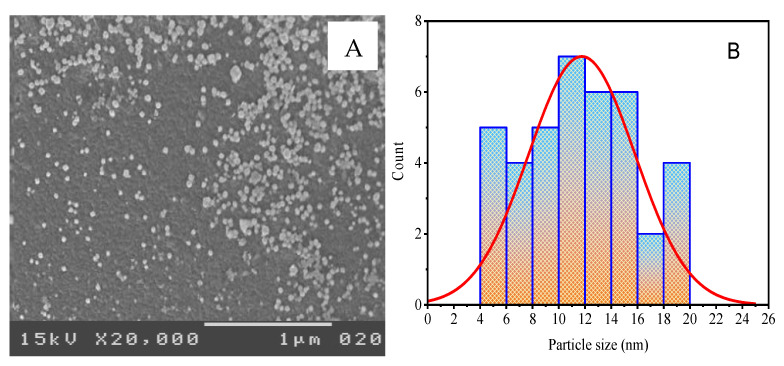
The as-biofabricated Ag NPs: (**A**) SEM image; (**B**) particle size distribution.

In general, the nanoparticles’ properties depended on their shape and size; smaller particles were higher in a specific surface area [68]. These properties consider the fulcrum to study for most nanoparticle applications, such as anticancer and antibacterial potentials. SEM morphologies of the biofabricated Ag NPs confirmed the results of XRD and TEM analyses, where they appeared as agglomerated spherical shapes (Figure 8). This agglomeration can be attributed to the bioactive constituents that are adsorbed onto the biofabricated Ag NPs [54,69]. Therefore, it strongly confirms the capping activity of the GB components that the formed Ag NPs surrounded. 

### 2.6. TEM Analysis

**Figure 9:**
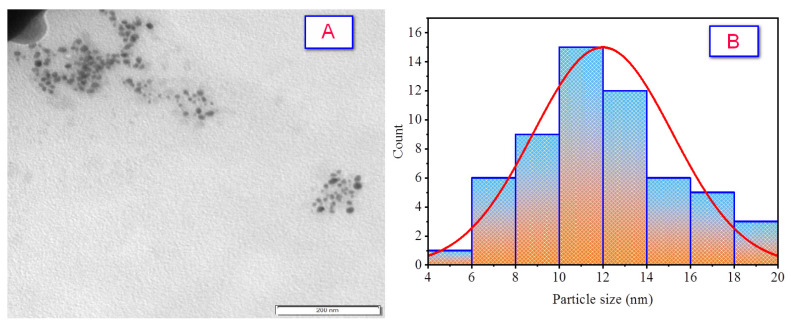
The as-biofabricated Ag NPs: (**A**) TEM image; (**B**) the distribution of particle size.

The TEM image of the biofabricated Ag NPs (Figure 9A) depicted spherical shapes that appeared as polycrystalline particles without intensive agglomeration. The Ag NPs after estimation and calculation were 11.99 ± 3.18 nm, showing a minimum particle size of 5.46 nm and a maximum particle size of 19.40 nm with a median of 11.81 nm, as the corresponding histogram form of the distribution particle size shows in Figure 9B. The finding was compatible with XRD results that confirmed the formation of the NPs at the nanoscale.

### 2.7. Zeta Potential

**Figure 10:**
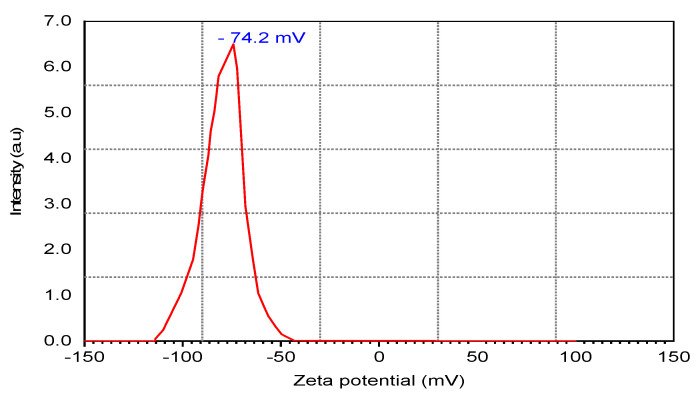
Zeta potential data of bio–fabricated Ag NPs using GB leaf extract.

Measurement of zeta potential estimates the suspended stability of NPs [70,71]. In Figure 10, zeta potential values of Ag NPs suspension stability were found to be −74.2 ± 2.45 mV, meaning that the NPs had evaded the agglomeration and excellent stability [70,72]. The biocomponents’ capping activity may bring negative potential value in GB leaf extracts. The colloidal particles in suspension were highly stable [70,72] when their charged surface passed the critical value ± 30 mV.

A few research groups have reported the as-fabricated Ag NPs so far, shown in Table 4, such as biofabricated Ag NPs using sugarcane leaves extract, by Srikhao et al. [73] and using leaves of Acacia melanoxylon as an effective bio-oxidizing/bioreducing agent, by Rajendrachari et al. [74]. While *Trichodesma indicum* leaf extract was reported by Kathiravan et al. [75], piper longum catkin extract was used as a reducing agent by Jayapriya et al. [76]. 

### 2.8. Anti-Coronaviruses Activity

Some studies demonstrated the effectiveness of various natural components and nanoparticles as treatment options for severe disorders. Nevertheless, only a small number of them have been used for therapeutic against viruses. A logical hypothesis would be to begin researching the properties and advantages of GB extract and Ag NPs as an antiviral versus the MERS-CoV and HCoV-229E based on our prior work on the conjugation of GB extract as a natural product and Ag NPs as an environmental nanoparticle synthesis [12,93].

**Figure 11:**
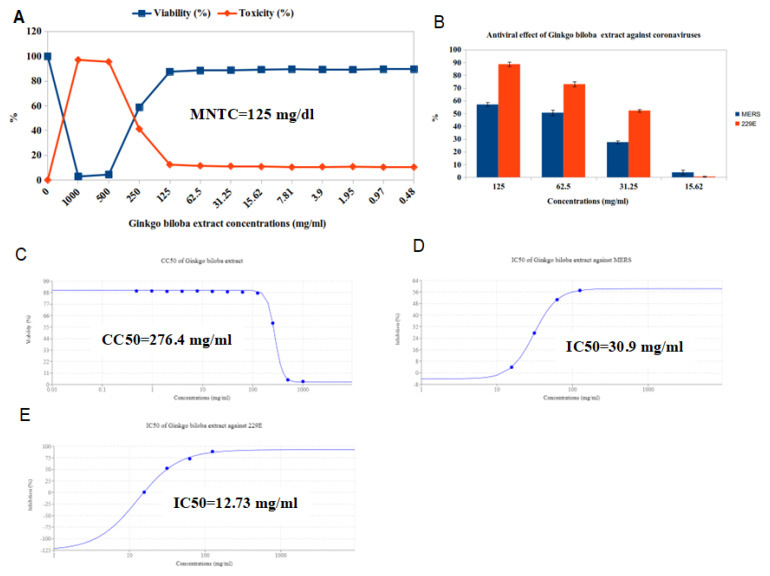
The anti–coronavirus activity of GB leaves extracts against MERS and 229E viruses using Vero cell lines. (**A**) Cytotoxicity; (**B**) antiviral activity; (**C**) CC50; (**D**) IC50 of the extract against MERS; (**E**) IC50 of the extract against 229E.

**Figure 12:**
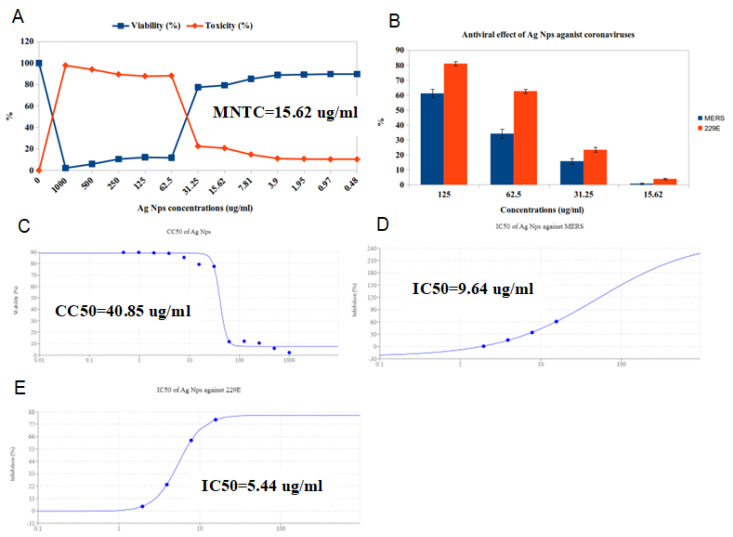
Anti–coronavirus activity of free silver nanoparticles (Ag NPs) against MERS and 229E viruses using the Vero cell line. (**A**) cytotoxicity; (**B**) antiviral activity; (**C**) CC50; (**D**) IC50 of the extract against MERS; (**E**) IC50 of the extract against 229E.

The GB extract and Ag NPs were evaluated by twofold dilutions in MEM with FCS by preparing twelve concentrations of it, beginning with 1000 mg/mL followed by 500 mg/mL, 250 mg/mL, 125 mg/mL, 62.5 mg/mL, 31.25 mg/mL, 15.62 mg/mL, 17.81 mg/mL, 3.9 mg/mL, 1.95 mg/mL, 0.97 mg/mL, and 0.48 mg/mL; and 1000 µg/mL followed by 500 ug/mL, 250 µg/mL, 125 µg/mL, 62.5 µg/mL, 31.25 ug/mL, 15.62 µg/mL, 17.81 µg/mL, 3.9 µg/mL, 1.95 ug/mL, 0.97 µg/mL, and 0.48 µg/mL. The cytotoxicity of GB extract and Ag NPs was evaluated on VERO cells using the MTT assay to ensure that the measured GB extract and Ag NPs doses were not harmful. The cytotoxicity concentrations (CC50) of GB extract and Ag NPs were 276.4 mg/mL and 40.85 µg/mL, while MNTC was 125 mg/mL and 15.62 mg/mL, respectively (Figure 11A,C and Figure 12A,C).

The antiviral activity of the GB extract against MERS and HCoV-229E viruses was investigated using the MTT antiviral test methodology. The results revealed that 125 mg/mL was the best concentration of GB extract against viruses because it reduced MERS replication by 57.09% and HCoV-229E by 88.66%. (Figure 11B). In addition, the finding was that Ag NPs have antiviral activity against MERS and HCoV-229E viruses, where the best concentration was 15.62, reducing the MERS replication by 61.09% and HCoV-229E by 81.05% (Figure 12B). It is generally known that the virus kills before infecting host cells when the IC50 concentration decreases more than the CC50 concentration.

Our results indicated that GB extract has moderate antiviral activity against MERS-CoV with an IC50 (30.9 mg/mL) lower than the CC50 of 276.4 mg/mL and SI of 8.94 (Table 5 and Figure 11C,D), and also has promising antiviral activity against HCoV-229E, with an IC50 of 12.73 mg/mL and SI of 21.71 (Table 5 and Figure 11C,E). From the results, we found that Ag NPs have mild antiviral activity versus MERS-CoV, with an IC50 (9.64 mg/mL) lower than the CC 50 (40.85 mg/mL) and SI of 4.23 (Table 5 and Figure 12C,D), and they also have moderate antiviral activity versus HCoV-229E, with an IC 50 (5.44 mg/mL) and SI of 7.51 (Table 5 and Figure 12C,E).

There are few treatment alternatives for treating coronaviruses in clinical settings. In order to successfully combat coronaviruses, medicinal chemists have made considerable efforts to find effective medicines for preventing coronavirus replication by focusing on several established therapeutic targets [94]. GB extract, a widely used plant in both Western and Eastern nations, has been extensively utilized to prevent and treat a variety of human syndromes, including heart problems, lung diseases, and central nervous system diseases [72,95,96]. Growing data suggest that several key components of GB extract, such as bioflavonoids, have broad antiviral properties against various DNA (such as human CMV virus) and RNA (such as Ebola virus, HIV, and SARS-CoV) viruses [97,98,99]. 

Our results indicated that GB extract could inhibit coronavirus (MERS and Hcov-229E) with SI of 8.94 and 21.71, respectively. We suggest this because GB has Ginkgolic acid (GA). Previous research claimed that the first virus-to-cell fusion event and the propagation of cell-to-cell infection are both inhibited by Ginkgolic acid’s antiviral mode of action [99]. We also showed that GA has broad-spectrum antiviral action against all three classes of recombinant protein, which might explain its ability to inhibit coronavirus Spike protein, a class I recombinant protein implicated in cell entry [100]. Furthermore, according to our findings, GA pre- or post-treatment reduces the generation of viral offspring. GA prevents the formation of viral proteins, as we previously found [101]. According to other studies, inhibiting viral DNA and protein synthesis may be GA’s secondary mode of action, which explains the virus’s powerful and effective suppression of HCV-229E and its ability to prevent SARS-CoV-2 infection [98,102,103,104,105]. 

The finding is that Ag NPs have mild antiviral activity versus MERS-CoV, with an SI of 4.23, but have moderate antiviral activity versus HcoV-229E, with an SI of 7.51. Ag NPs may interact with the viral surface as a potential antiviral mechanism, destroying viral genomic material or preventing it from entering the cell membrane. To prevent the virus from interacting with the cell membrane, Ag NPs additionally adhere to the viral entity. Ag NPs, likewise, inhibit the viral entity’s nucleocapsid inside the cell. In Moro, the Ag NPs bind to viral genomic material, preventing host cell genome replication. Last, but not least, biological processes such as protein synthesis are stopped to prevent the viral entity from replicating [106].

An earlier study revealed that compared to chemical- or antibody-based antiviral therapies, the future use of nanoparticles as innovative antiviral treatments exhibited a decreased chance of developing drug resistance [107]. Furthermore, our findings supported using Ag NPs as virucidal agents on fomites by demonstrating their safety. However, more toxicity research has to be carried out for future therapeutic applications [108].

## 3. Materials and Methods

### 3.1. Materials

In the current work, all chemical substances used were analytical grade. For HPLC analysis, we used methanol (purity 99.9%) from Merck, UK, H_3_PO_4_ acid, purity 85%, NaH_2_PO_4_, HCl acid, purity 37%, and NaOH (Scharlau, Spain). The buffer solution was prepared by weighing about 16.8 g of NaH_2_PO_4_ and 0.5 mL of H_3_PO_4_ acid 85% in 700 mL of deionized water. Ginkgo leaves were collected from a tree growing in Wuhu, Anhui, China, in the beginning of the summer season in 2020. The species was authenticated at Al-Azhar University, Assuit, Egypt (Botany and Microbiology Department, Faculty of Science).

### 3.2. The Active Compounds of Ginkgo Leaves Extract

Plant materials (Ginkgo leaves) were dried to a constant weight at room temperature and ground into a powder. Five grams of plant powder were homogenized, then macerated in a stoppered container with 100 mL methanol (99.9%) and allowed to stand at room temperature for 24 h. The extract and powder were placed in a sonicator at 40 °C for 60 min for conventional extraction. Then this extract was filtered and concentrated under vacuum at 40 °C by using a Rota vapor to provide crude extract.

### 3.3. Total Phenolics Content

The Folin–Ciocalteu technique was applied to quantify the total amount of phenolics [109,110]. A volume of 3 mL of Folin–Ciocalteau (10%) was mixed with 0.8 mL sodium bicarbonate (7.5%) and 0.05 mL plant extract. For 30 min, the reactants were incubated at room temperature. A spectrophotometer measured the absorbance at 765 nm (Milton Roy, Spectronic 1201). The data for phenolics were given as mg gallic acid equivalents/g dry extract. 

### 3.4. Total Flavonoids Content 

According to Chang et al. [111], total flavonoids were determined. A total of 0.1 mL extract was added to 3.90 mL distilled H_2_O and 0.3 mL sodium nitrite (5%) solution; this mixture reacted for 5 min. Then, 0.3 mL aluminum chloride (10%) solution was added and left to react for 6 min. Afterward, this solution was treated with 2 mL of 1 M sodium hydroxide. Finally, distilled water was added to a constant volume in all samples. The absorbance was detected at 510 nm against a blank using the previously mentioned spectrophotometer. Flavonoids were calculated as mg quercetin equivalents/g dry extract.

### 3.5. HPLC Analysis

The plant extract was diluted with acidic methyl alcohol (HPLC analytical grade) and clarified using a 0.22 μm Teflon syringe filter (Cameo, MN). The separation of phenolic acids was employed through a mobile phase comprising two solvents: 0.1% methanol and phosphoric acid (50:50  *v*/*v*). The flow rate was attuned to 1.0 mL/min; the sensor was set at 280 nm with the mobile phase [112]. For flavonoids, the mobile phase involved a binary mixture of methanol/water (50:50 *v*/*v*) with a pH adjusted to 2.8 using phosphoric acid at an isocratic flow rate of 1.0 mL min^−1^ [113].

### 3.6. The Biogenic Synthesis of Ag NPs

Firstly, Ginkgo leaves were washed well with deionized water, dried, and pulverized through a 50-mesh sieve. Two grams of the obtained Ginkgo leaf powder was dissolved in 100 mL deionized water and placed in an ultrasonic bath at 80 °C for 40 min. After cooling, aqueous extracts of the GB were obtained by centrifugation at 3000 g for 30 min. The aqueous GB leaf extracts were treated with the AgNO_3_ solution “5% *w*/*v* in deionized water”, drop by drop, at a ratio of 1:2, at the reaction temperature of 80 °C, 3000 rpm, for one hour, to produce the highest yield of biogenic Ag NPs. After being in the dark for 24 h, the biosynthesized NPs were mixed, and the resulting product was centrifuged five times for ten minutes at 3000 rpm. The resulting NPs were heated for two hours at 100 °C after being centrifuged three times with deionized water at a 3000 rpm speed. The ultimate result was a dark brown, demonstrating the production of Ag NPs and regular monitoring of the solution’s color change.

### 3.7. Characterization

Ginkgo leaf extracts were analyzed using HPLC (Agilent 1100), which consists of two LC-pump pumps (Shimadzu, Japan) and a UV–Vis detector, with a C18 column (particle size: 5 m, 125 mm, 4.60 mm). The Agilent Chem-Station was used to collect and analyze chromatograms. The Ag NPs analyses were conducted via different instrumental analysis tools, using the digital balance of 5 digits Citizen [CX 265]. UV–Vis absorption spectra of the Ag NPs were measured using PerkinElmer (Lambda-750-UV/Vis/Nir) spectrophotometer equipped with 1 cm quartz cells at room temperature. The particle size of Ag NPs was investigated by a Philips X-ray diffractometer (PW 1710). A Nicolet iS10-FT-IR spectrometer was used for FT-IR analysis of Ag NPs in a wavenumber range 400–4000 cm^−1^. The size and morphology of the Ag NPs were studied using scanning electron ((SEM; JEOL (JSM 5400LV)) and transmission electron ((TEM; JEOL (JEM-100 CXII)) microscopy.

### 3.8. Cytotoxicity of Ginkgo Biloba Extract and Ag NPs on VERO Cells and Viruses

Utilizing the MTT test, antiviral activity was assessed [114]. All viruses and VERO cells (ATCC: CCL-81) were acquired from VACSERA Research Foundation, Egypt. Through twofold dilutions in MEM with FCS, starting with 1000 to 0.48 mg/mL and 1000 to 0.48 µg/mL, we assessed the maximum nontoxic concentration (MNTC) of each GB extract and Ag NPs. The growth media of microtiter plates was decanted, and a range of GB extract and Ag NPs concentrations were created. After VERO cells were washed twice with wash media and the monolayer was formed, double-fold dilutions of the test sample were prepared in the minimal necessary medium. Three wells were left as controls, and 0.1 mL of each dilution was incubated in a separate well. We looked at the physical toxicity traits in the cells, such as the monolayer’s complete or partial loss, cell granulation, rounding, or shrinkage. The preparation of MTT solution was achieved by using PBS at 5 mg/mL (BIO BASIC CANADA INC). The MTT solution (20 µL) was added to each well and mixed with the medium by shaking each well at 150 rpm for five minutes. The medium was withdrawn after the MTT was digested at (37 °C, 5% CO_2_) for 1–5 h (if necessary, the plate was dried on paper towels). To effectively integrate the formazan and solvent, the formazan was resuspended in 200 µL of DMSO and shaken at 150 rpm for five minutes. At 560 nm, the optical density was calculated, and at 620 nm, the background was removed. Optical density and cell count ought to be vital to connect. Each extract’s maximum nontoxic concentration (MNTC) was determined and used for additional biological studies [114,115].

### 3.9. MTT Assay Protocol

A 96-well plate with 10,000 cells plated in two hundred µL of medium per well was used to measure the antiviral activity [116]. After incubating the virus suspension and equal volumes (1:1 *v*/*v*) of the tested sample for one hour, one hundred µL of the viral/sample suspension was added. The mixture was shaken at 150 rpm for 5 min. The three wells for blank controls were left empty. The remaining wells were incubated (at 5%, CO_2_, 37 °C) to allow the cells to attach to the wells overnight. The viral/sample suspension was incubated (at 5% CO_2_, 37 °C) for one day to give the virus time to work. Next, 20 mL of MTT solution was added to each well of 96-well plates, which should have at least 2 mL of MTT solution per well. The MTT solution was thoroughly mixed with the medium by shaking the plates at 150 rpm for 5 min. Plates were incubated for 1–5 h (at 37 °C, 5% CO_2_) to digest MTT; medium was removed (plates were dried on paper towels to remove residue). Next, formazan (MTT metabolic product) was resuspended in 200 µL DMSO and shaken at 150 rpm for 5 min. The optical density was determined at 620 nm or 560 nm, and the background was subtracted. The link between optical density and cell number ought to be straightforward [114,115].

According to Pauwels et al. [117], the percentage of antiviral activity of the compounds evaluated was determined using the formula below.

Antiviral activity = [(mean optical density of cell controls − mean optical density of virus controls)/(optical density of the test mean optical density of virus controls)] × 100%. The CC_50_ and IC_50_ were calculated using the IC_50_ online calculator server (https://www.aatbio.com/tools/ic50-calculator, accessed on 25 April 2022) [118].

## 4. Conclusions

The current study demonstrated the antiviral activity of *Ginkgo biloba* leaves extract and silver nanoparticles against MERS-CoV and HCoV-229E viruses in vitro. The finding was that GB leaves extract had promising and moderate antiviral activity against MERS-CoV and HCoV-229E virus, respectively, when compared with the silver nanoparticles effect, that had mild activity. The finding was that when using HPLC, the most common secondary metabolites were flavonoids such as apegenin, luteolin, myricetin, and catechin, and phenolic compounds such as pyrogallol, caffeic acid, gallic acid, and ellagic acid. We also used X-ray diffraction, TEM, XRD, FTIR, and UV–visible spectroscopy techniques to characterize silver nanoparticles, which indicated a range size from 5.46 to 19.40 nm and an average particle diameter of 11.81 nm.

## Figures and Tables

**Table 1:** Total contents of flavonoids and phenolics (mg/g dry weight) of GB leaves.

Sample	Contents (mg/g)	Methanol (70%)	Acetone (70%)	Ethanol (70%)	Water
GB leaves	Total flavonoids	49.56 ± 2.82	54.59 ± 1.99	39.16 ± 2.12	27.55 ± 2.13
	Total phenolics	53.28 ± 3.94	61.22 ± 3.19	33.38 ± 2.04	39.17 ± 2.47

**Table 2:** HPLC flavonoids data of *G. biloba* leaves.

RT	Compound	Concentration (μg/mL)
6.0	Apegenin	5.03
9.0	Luteolin	3.02
11.0	Myricetin	3.88
12.0	Catechin	11.69

**Table 3:** HPLC chromatogram of phenolics from GB leaves.

RT	Compound	Concentration (μg/mL)
3.0	Pyrogallol	1.88
8.0	Caffeic acid	0.45
10.0	Gallic acid	15.27
15.0	Ellagic acid	2.42

**Table 4:** The biofabricated Ag NPs using some plant sources with particle sizes and shapes.

Plant	Particle Size (nm)	Shape	Reference
*Citrullus lanatus fruit rind*	17.96 ± 0.16 nm	Spherical	[77]
*Pedalium murex leaf*	14 nm	Cubic	[50]
*Nigella arvensis seed*	8.5 nm	Spherical	[78]
*Bacillus species*	10 nm	Spherical, rod, and octagonal	[79]
*Trichoderma*	10 nm	Spherical	[80]
*longibrachiatum*	10–16 nm	Spherical	[7]
*Ginkgo biloba leaf*	40.2 ± 1.2 nm	Spherical or oval	[81]
*Entada spiralis*	18.49 ± 4.23 nm	Spherical	[82]
*Tropaeolum majus leaf*	35–55 nm	Round	[83]
*Morus nigra leaves*	23 nm	Cubic	[84]
*Pithecellobium dulce leaves*	62 nm	Spherical rods	[85]
*Allium giganteum shoots*	12 nm	Spherical	[86]
*Piper betle leaves*	3–37 nm	Spherical	[87]
*Allium sativaum root*	7.3 nm	Spherical	[88]
*Annona reticulate leaves*	6–8 nm	-	[89]
Salvia officinalis leaf	41 nm	Spherical	[90]
Rumex dantatus root	25–70 nm	-	[91]
Spinacia oleracea leaves	15 ± 5 nm	Cubic	[92]
*Ginkgo biloba leaves*	11.99 ± 3.18	Spherical	This study

**Table 5:** Antiviral activity of GB leaves extract and Ag NPs anti human coronavirus (MERS-CoV and 229E).

Treatment Compounds	Virus	MTNC	CC _50_	IC _50_	SI.
GB leaves extract (mg/mL)	MERS			30.9	8.94
	229E	125	276.49	12.7	21.76
Ag NPs (µg/mL)	MERS			9.3	4.39
	229E	15.62	40.85	7.01	5.82

## Data Availability

Not applicable.

## Sample Availability

The compounds in this paper are available from the authors on reasonable request.
